# Brown adipose tissue activity as a target for the treatment of obesity/insulin resistance

**DOI:** 10.3389/fphys.2015.00004

**Published:** 2015-01-30

**Authors:** Anne-Laure Poher, Jordi Altirriba, Christelle Veyrat-Durebex, Françoise Rohner-Jeanrenaud

**Affiliations:** ^1^Laboratory of Metabolism, Department of Internal Medicine Specialties, Faculty of Medicine, University of GenevaGeneva, Switzerland; ^2^Department of Cell Physiology and Metabolism, University of GenevaGeneva, Switzerland

**Keywords:** UCP1, FGF21, BMP, PTEN, obesity, diabetes, IL-6, gestation

## Abstract

Presence of brown adipose tissue (BAT), characterized by the expression of the thermogenic uncoupling protein 1 (UCP1), has recently been described in adult humans. UCP1 is expressed in classical brown adipocytes, as well as in “beige cells” in white adipose tissue (WAT). The thermogenic activity of BAT is mainly controlled by the sympathetic nervous system. Endocrine factors, such as fibroblast growth factor 21 (FGF21) and bone morphogenic protein factor-9 (BMP-9), predominantly produced in the liver, were shown to lead to activation of BAT thermogenesis, as well as to “browning” of WAT. This was also observed in response to irisin, a hormone secreted by skeletal muscles. Different approaches were used to delineate the impact of UCP1 on insulin sensitivity. When studied under thermoneutral conditions, UCP1 knockout mice exhibited markedly increased metabolic efficiency due to impaired thermogenesis. The impact of UCP1 deletion on insulin sensitivity in these mice was not reported. Conversely, several studies in both rodents and humans have shown that BAT activation (by cold exposure, β3-agonist treatment, transplantation and others) improves glucose tolerance and insulin sensitivity. Interestingly, similar results were obtained by adipose tissue-specific overexpression of PR-domain-containing 16 (PRDM16) or BMP4 in mice. The mediators of such beneficial effects seem to include FGF21, interleukin-6, BMP8B and prostaglandin D2 synthase. Interestingly, some of these molecules can be secreted by BAT itself, indicating the occurrence of autocrine effects. Stimulation of BAT activity and/or recruitment of UCP1-positive cells are therefore relevant targets for the treatment of obesity/type 2 diabetes in humans.

## Introduction

Obesity, well known to be associated with a number of comorbidities, including insulin resistance and type 2 diabetes, has become a major public health problem in recent decades, and has reached epidemic proportions, not only in high-income countries, but also in most middle-income societies. It is defined as an accumulation of adipose tissue that is of sufficient magnitude to impair health (WHO, 2014). Excess weight is usually defined by the body mass index or BMI. The normal BMI range is 18.5–25 kg/m^2^, although the range may vary for different countries. Individuals with a BMI above 30 kg/m^2^ are classified as obese; those with a BMI between 25 and 30 kg/m^2^ are considered to be overweight. In general, the term obesity applies to both the obese and the overweight subjects. More than the total body weight, the distribution of the stored fat is of importance for the development of obesity and its comorbidities. Thus, central or visceral obesity, in which fat accumulates in the trunk and in the abdominal cavity (in the mesentery and around the viscera), is associated with a much higher risk for several diseases than excess subcutaneous fat accumulation. Obesity has profound effects on tissue insulin sensitivity, and therefore on systemic glucose homeostasis. Insulin resistance is present even in simple obesity, without hyperglycemia, indicating a fundamental abnormality of insulin signaling in states of excess adipose tissue mass. The epidemiologic association of obesity, particularly of the visceral type, with type 2 diabetes has been recognized for decades. According to the World Health Organization, 347 millions of people are diabetic in the world, and it is predicted that in 2030, diabetes will be the 7th cause of death considering the worldwide population (WHO, 2014).

Although the pathogenesis of obesity is extremely complex and is far from being unraveled, the key component of the obesity epidemic is long-term dysregulation of energy balance, comprising increased energy intake and/or reduced energy expenditure. Despite active research and impressive improvements in the understanding of the regulation of energy balance, there are only a very limited number of drugs that can be used for the efficient treatment of obesity and its comorbidities. Targeting specific components of the neuroendocrine regulation of energy intake, such as leptin or hypothalamic neuropeptides, has disappointingly revealed unsuccessful as yet. New alternatives focusing on adipose tissue function could potentially be of therapeutic relevance in the future.

Two different types of adipose tissue have been described: brown adipose tissue (BAT), composed mainly of brown adipocytes, and white adipose tissue (WAT), defined by a majority of white adipocytes, both tissues being able to accumulate lipids in intracellular droplets. WAT is an energy-storing tissue that has evolutionary enabled humans to survive for longer periods between meals, storing energy mainly as triglycerides and releasing fatty acids during fasting periods. In recent times, when food has become cheaper and more widely available, excessive WAT storage contributed to the worldwide alarming development of obesity mentioned above (World Health Organization, 2009). White adipocytes are composed of a large single, spherical lipid vacuole and a peripherally located nucleus, together with few mitochondria. WAT has endocrine activity, secreting several factors and hormones, such as leptin and adiponectin. Under certain conditions, another type of adipocytes, named brite or beige cells, can be found dispersed within some of the WAT depots. These cells, which will be discussed below, present a phenotype with metabolic properties that are closest to brown than to white adipocytes.

BAT consists in brown adipocytes, characterized by multiple, small, multilocular lipid droplets with a central nucleus and a high number of mitochondria. BAT is a highly vascularized tissue innervated by the sympathetic nervous system. The mitochondria of BAT are characterized by the presence of uncoupling protein-1 (UCP1) in the inner mitochondrial membrane. When activated, this protein uncouples mitochondrial respiration from ATP synthesis, resulting in heat production, a process that consumes substantial amounts of fuels. BAT, the principal effector organ of non-shivering thermogenesis (i.e., heat production that does not involve skeletal muscle contraction), is present in most mammals and its maturation in the perinatal period varies between species, according to their developmental status at birth (Tews and Wabitsch, 2011).

In humans, BAT develops in the fetus during gestation. Thus, the amount of UCP1 increases during fetal development, peaks at birth, before declining over the first 9 months (Lean et al., 1986; Tews and Wabitsch, 2011). The notion that human BAT is solely apparent during the neonatal stage prevailed for decades (Heaton, 1972; Nedergaard et al., 2007). In 2009, functional human BAT was identified in adults by a combination of CT scans and fluorodeoxyglucose positron emission tomography (FDG-PET) (Cypess et al., 2009; van Marken Lichtenbelt et al., 2009; Virtanen et al., 2009). The areas in which BAT is observed in adult humans include supraclavicular, neck, paravertebral, and suprarenal sites (Nedergaard et al., 2007). Estimates of BAT mass and activity from FDG-PET studies suggest that humans have, on average, 50–80 g of BAT (Peirce et al., 2014). Quantitatively, it was estimated that 50 g of BAT can burn as much as 20% of daily energy intake (Rothwell and Stock, 1983). As an example, in a subject with 63 g of supraclavicular BAT, it was calculated that if the depot was fully activated, it would burn an amount of energy equivalent to 4.1 kg of WAT (Virtanen et al., 2009). It can therefore be concluded that, even though the BAT depots are present in small amounts, the activated tissue has the potential to substantially contribute to energy expenditure (Nedergaard et al., 2007).

BAT activity is well known to mostly rely on lipid metabolism, UCP1 being directly activated by fatty acids (Cannon and Nedergaard, 2004). Along this line, it was recently demonstrated that chronic activation of the β3 adrenoreceptor induces coupled increases in lipolysis, *de novo* lipogenesis and fatty acid beta-oxidation not only in white, but also in BAT (Mottillo et al., 2014). Thus, the continuous cycling of triglyceride hydrolysis coupled to resynthesis, which requires large amounts of ATP, could be another important mechanism to increase thermogenesis in BAT, in addition to the role of UCP1 activation in this process (Mottillo et al., 2014). Similarly to what was proposed for skeletal muscle (Dulloo et al., 2004), this may contribute to dissipate excess lipids as occurs during prolonged stimulation of lipolysis (e.g., chronic β3 adrenoreceptor treatment).

In addition to using lipids, BAT also displays a very high rate of glucose uptake, particularly under sympathetic activation (Cannon and Nedergaard, 2004). Interestingly, BAT glucose uptake is close to the values observed for metastasis in cancer in humans (Aukema et al., 2010). This tissue also responds to insulin with a 5-fold increase in glucose uptake, without any change in blood flow (Orava et al., 2011), while under cold exposure, glucose uptake increases by 12-folds, dissipating energy as a function of increased blood flow (Orava et al., 2011). Regarding the fate of glucose in brown adipocytes under anabolic conditions characterized by high insulin levels, it is essentially metabolized to provide glycerol-3-phosphate for triglyceride synthesis or acetyl- CoA for *de novo* fatty acid synthesis (Cannon and Nedergaard, 2004).

To investigate the role of BAT, of UCP1 in particular, studies were carried out in UCP1 knockout mice. Surprisingly, no particular phenotype was noted in these mice when they were kept at 23°C, except for their increased cold sensitivity (Enerback et al., 1997; Kontani et al., 2005). In contrast, when bred under thermoneutral conditions (29°C), UCP1 knockout mice exhibited markedly enhanced metabolic efficiency due to impaired thermogenesis (Feldmann et al., 2009).

Altogether, the existing literature suggests that BAT activation is not only involved in non-shivering thermogenesis, but also in the regulation of insulin-mediated glucose disposal. Whether brown and brite adipocytes display some degree of specialization with regard to these different functions has to be established. The aims of this review are to describe some of the main factors regulating UCP1 activity in brown and brite adipocytes, as well as to discuss the potential role of UCP1 activation for the treatment of insulin resistance and type 2 diabetes associated with obesity.

## Factors affecting BAT function and energy metabolism improve overall metabolism

In rodents, brown adipocytes are found in discrete areas, such as interscapular, cervical, peri-aortic, peri-renal, intercostal and mediastinal depots (Cinti, 2001), which are referred to as “classical” BAT depots. In addition, brown adipocytes can be found scattered in WAT, especially upon cold exposure (Young et al., 1984; Guerra et al., 1998), treatment with β-adrenergic (Himms-Hagen et al., 2000), or with peroxisome-proliferator-activated receptor-γ (PPAR-γ) agonists (Petrovic et al., 2010). These brown-like adipocytes have interchangeably been called “recruitable” (Tseng et al., 2008; Schulz et al., 2013), “beige” (Ishibashi and Seale, 2010; Auffret et al., 2012; Wu et al., 2012), or “brite” (for brown to white) (Petrovic et al., 2010; Gburcik et al., 2012) cells.

Lineage-tracing studies showed that brown adipocytes in classic BAT areas derive from myogenic factor 5 (*Myf* 5^+^)- positive progenitor cells, similarly to skeletal myocytes (Timmons et al., 2007). In contrast, “brite” adipocytes have been shown to originate from Myf-negative (*Myf* 5^−^) progenitor cells, much like white adipocytes (Petrovic et al., 2010; Long et al., 2014). Whether “brite” adipocytes descend from unique precursors, or share progenitors with either white or classic brown adipocytes still remains to be established (for rev., see Chechi et al., 2013). Interestingly, the “browning” of WAT (i.e., increased proportion of brown adipocytes) may also involve transdifferentiation of white-to-brown adipose cells (Smorlesi et al., 2012; Frontini et al., 2013), although this issue is still a matter of debate (Wu et al., 2012).

Whatever their developmental origin, white, “brite” and brown adipocytes seem to greatly differ in their function. As mentioned above, BAT is the effector organ of non-shivering thermogenesis (both cold and diet-induced) that, by utilizing large quantities of glucose and lipids from the circulation, can promote negative energy balance. Moreover, the role of BAT activation appears to be broader than solely the promotion of negative energy balance (for rev., see Peirce and Vidal-Puig, 2013). Indeed, BAT is now known to exert anti-type 2 diabetic effects associated with improvments of dyslipidemia and insulin secretion as well as decrease insulin resistance in type 2 diabetes (de Souza et al., 1997; Liu et al., 1998; Frontini et al., 2013; Peirce and Vidal-Puig, 2013). These effects are partly interrelated, but can also be dissociated and exerted by different UCP1-expressing types of adipocytes (i.e., brown and “brite” adipocytes). However, as these different cells are often mixed, such as occurs for classical and “brite” adipocytes in some human depots (Wu et al., 2012; Cypess et al., 2013; Jespersen et al., 2013), only the use of specific cell surface markers (i.e., ASC-1, PAT2, and P2RX5 for white, “brite” and brown adipocytes, respectively) will allow for their identification, as well as for the precise understanding of their respective therapeutic properties (Ussar et al., 2014).

BAT activation by cold exposure, β3-agonist or thyroid hormone treatment was shown to improve glucose tolerance and insulin sensitivity (Cawthorne et al., 1984; Forest et al., 1987; Peirce and Vidal-Puig, 2013). Similar observations were obtained by adipose tissue-specific overexpression of PR-domain-containing 16 (PRDM16) in mice. This Zinc-finger transcription factor induces differentiation of brown adipocytes (Seale et al., 2011). The main mediators of such beneficial effects seem to include fibroblast growth factor 21 (FGF21), interleukin-6 (IL-6), bone morphogenic proteins (BMPs) and prostaglandin D2 synthase. Interestingly, some of these molecules, called batokines, can be secreted by BAT itself, indicating the occurrence of autocrine effects.

Fibroblast growth factor 21 is a member of the fibroblast growth factor (FGF) family that acts as a hormone and that, in contrast to other endocrine FGFs, is devoid of proliferative activity (Itoh, 2014). It is expressed in BAT and WAT, although its main production site is the liver (Nishimura et al., 2000; Muise et al., 2008; Schulz et al., 2013; Zafrir, 2013). Tissue-specific FGF21 regulation was shown to occur in response to chronic cold exposure in mice (Fisher et al., 2012). Under this condition, FGF21 expression was indeed decreased in the liver, but enhanced in BAT, as well as in WAT, where it acted to markedly increase UCP1 expression and the “browning” of subcutaneous tissue (Fisher et al., 2012). Interestingly, in humans, a mild cold exposure (12 h to 19°C) was recently shown to increase the diurnal plasma FGF21 levels, with a positive correlation with the changes in adipose tissue microdialysate glycerol and total energy expenditure (Lee et al., 2013). This suggested that FGF21 could play a similar role in humans as in rodents in promoting cold-induced metabolic changes (i.e., lipolysis and cold-induced thermogenesis). In adipose tissue, it appears that PPARγ transcriptionally controls FGF21, which then acts as an autocrine or paracrine way to increase PPARγ transcriptional activity in a feed-forward loop system (Wang et al., 2008; Dutchak et al., 2012). FGF21 deficiency in mice was shown to result in impaired ability to adapt to long-term cold exposure with diminished “browning” of WAT (Fisher et al., 2012). At the opposite, systemic administration of FGF21 in obese mice resulted in reduced adiposity, improved glycemic control, as well as increased energy expenditure, as mentioned by the authors (Coskun et al., 2008). Altogether, these observations suggest that FGF21 may be a key factor linking UCP1 expression to improved glucose metabolism. It may also exert determinant developmental effects, given the observation that the postnatal maturation of BAT appears to relate to the onset of feeding and initiation of hepatic function, as mediated by the release of FGF21 (Hondares et al., 2010). In addition, it was recently proposed that FGF21 could act within the central nervous system, both at the level of the hypothalamus and the hindbrain to promote a set of responses that occur during starvation (i.e. increase in corticosterone levels, suppression of physical activity, alteration in circadian behavior) (Bookout et al., 2013). This raises the possibility that, in contrast to its beneficial effects on peripheral metabolism, FGF21 may exert deleterious effects by acting centrally.

Interleukin-6 (IL-6), predominantly known as a pro-inflammatory cytokine, is secreted by skeletal muscle (Pal et al., 2014), helper T cells, as well as by WAT and BAT (Mohamed-Ali et al., 1997; Cannon et al., 1998). Several studies implicated IL-6 as a co-inducer of the development of obesity-associated insulin resistance preceding the onset of type 2 diabetes (Pal et al., 2014). This is in keeping with the observation of increased plasma IL-6 levels in obese patients (Cottam et al., 2004). In such patients, IL-6 is preferentially secreted from visceral rather than from subcutaneous adipocytes and may participate in the prevailing increase in sympathetic outflow by exerting central effects (Wallenius et al., 2002; Fain et al., 2004). Paradoxically, central IL-6 delivery was shown to suppress weight gain and visceral obesity, without affecting food intake (Li et al., 2002). The treatment also enhanced UCP1 protein levels in BAT, *via* stimulation of the sympathetic nervous system (Li et al., 2002). This was mediated by phosphorylation of the signal transducer and activator of transcription 3 (pSTAT3). Interestingly, chronic central IL-6 stimulation desensitized IL-6 signal transduction characterized by reversal of elevated pSTAT3 levels (Li et al., 2002). Such desensitization is likely occurring in situations of chronic elevation in IL-6 levels, such as occurs in human obesity. It should be added that the understanding of the role of IL-6 is more complex, as this cytokine is known to be secreted by skeletal muscle in response to exercise, exerting insulin sensitizing effects (Kelly et al., 2004; Petersen and Pedersen, 2005). Along this line, it was recently shown that BAT transplantation into the abdominal cavity of high fat diet-induced obese mice was able to improve their glucose tolerance, increase their insulin sensitivity, lower their body weight, decrease their fat mass and completely reverse their insulin resistance (Stanford et al., 2013). BAT transplantation also increased insulin-stimulated glucose uptake in BAT, WAT, and heart, but not in skeletal muscle (Stanford et al., 2013). Importantly, the improved metabolic profile was lost when BAT used for transplantation came from IL-6 knockout mice, clearly demonstrating that BAT-derived IL-6 is required for the profound effects of BAT transplantation on glucose homeostasis and insulin sensitivity (Stanford et al., 2013).

Apart from IL-6, another circulating factor, named irisin, was shown to be produced by skeletal muscles during physical exercise in rodents (De Matteis et al., 2013). Irisin, obtained by cleavage from the precursor protein, fibronectin type III domain containing 5 (FNDC5), was described as promoting the appearance/recruitment of “brite” cells in white adipose depots (Bostrom et al., 2012; Lee et al., 2014a). However, the existence of this protein and its role in humans is still a matter of debate (Elsen et al., 2014a).

Thyroid hormones (THs) are well known mediators of overall energy expenditure (Klieverik et al., 2009). Treatment with THs induces UCP1 expression in brown adipocytes in rats, following their binding to TH-responsive elements in the UCP1 promoter (Guerra et al., 1996). Type 2 iodothyronine deiodinase (D2), responsible for the transformation of thyroxine (T4) to triiodothyronine (T3), is also inducing UCP1 expression locally, in BAT (de Jesus et al., 2001). Furthermore, treatment of brown adipocytes and human skeletal myocytes with bile acids (BA) were shown to increase D2 activity and oxygen consumption *via* the activation of UCP1 (Watanabe et al., 2006). In both rodents and humans, this BA-D2-UCP1 pathway appears to be crucial for the fine-tuning of energy homeostasis, improving the metabolic control (Watanabe et al., 2006). Thyroid receptors (TRs) are composed of several isoforms that specifically regulate UCP1 expression and thermogenesis. The α isoform was shown to regulate adaptive thermogenesis, whereas the β isoform appears to modulate UCP1 expression, without increasing thermogenesis (Ribeiro et al., 2001). In humans, a unique patient suffering from extreme diabetes due to a mutation in the insulin receptor gene had to undergo total thyroidectomy because of the presence of a papillary carcinoma. Thirty months after the initial treatment of the thyroid cancer (radioiodine and levothyroxine), remarkable improvements in glycemia were noted and the anti-diabetic treatment could even be discontinued. A PET/CT study revealed the presence of BAT depots in the lower neck, suprascapular, mediastinal, and thoracic paravertebral regions. Interestingly, increased FDG uptake was also noted in the subcutaneous fat, in particular in the pelvic area and over the lower extremities. Overall, the sequence of events in this patient suggests that the metabolic and trophic effects of THs on BAT may play a critical role in non-insulin-mediated glucose utilization, ultimately leading to near-normal glucose levels (Skarulis et al., 2010).

Bone morphogenic proteins (BMPs) are members of the transforming growth factor β superfamily (TGF-β). They were originally thought to be factors inducing bone formation, but were then described to be involved in the development and function of many tissues, such as the intestine, brain and WAT (Hogan, 1996). Some members of the BMP family were shown to play a role in energy homeostasis and the early steps of adipogenesis, in particular. Among the 20 BMP family members, BMP-7 has been implicated in the development of BAT, being able to drive the complete brown fat differentiation program, including PRDM16 expression (Modica and Wolfrum, 2013). BMP-7 can also affect energy homeostasis by acting on mature brown adipocytes, resulting in the induction of UCP1, thereby enhancing thermogenesis. As it is not expressed in mature brown adipocytes, BMP7 appears to exert its action on BAT as an endocrine factor. In addition to its effect on BAT, BMP-7 was also reported to induce the “browning” of WAT and to improve insulin sensitivity (Schulz et al., 2011). Finally, several hypothalamic nuclei were shown to express BMP-7, suggesting that it may regulate BAT function *via* a central mechanism, also responsible for decreased food intake (Modica and Wolfrum, 2013).

BMP8B, another member of the BMP family, was found to be expressed in BAT, as well as in the hypothalamus (Contreras et al., 2014). Central administration of BMP8B induced thermogenesis and increased core temperature, leading to weight loss (Contreras et al., 2014). This effect, exerted within the ventromedial hypothalamus (VMH), was described as being AMPK-dependent, resulting in the activation of the sympathetic outlflow to BAT, without any change in the feeding behavior (Whittle et al., 2012; Contreras et al., 2014).

In contrast to BMP7 and BMP8B, BMP4 was shown to promote the differentiation of mesenchymal stem cells into white adipocytes, inducing fat storage and decreasing energy expenditure in rodents (Modica and Wolfrum, 2013; Contreras et al., 2014). However, in primary human adipose stem cells, both BMP4 and BMP7 induced a white-to-brown adipocyte transdifferentiation (Elsen et al., 2014b), pointing to the occurrence of different effects, depending on the model used for investigation. Further studies are needed to clarify and strengthen the role of BMP proteins in the regulation of BAT or “brite” cells and their consequences on metabolic homeostasis.

Phosphatase and tensin homolog deleted on chromosome ten (PTEN), a well-known tumor suppressor is a phosphatase that specifically catalyzes the dephosphorylation of phosphatidylinositol-3,4,5-triphosphate (PIP_3)_, in phosphatidylinositol-4,5-diphosphate PIP_2_ (Cantley and Neel, 1999). This enzyme counteracts the action of phosphatidylinositol-4,5-bisphosphate 3-kinase (PI3K), resulting in inhibition of the AKT signaling pathway involved in multiple biological processes, including insulin action. Activation of AKT is known to trigger a complex cascade of events that include the inhibition of FOXO transcription factors (Ortega-Molina et al., 2012). Interestingly, mice carrying additional copies of *Pten* (Pten^tg^ mice) are not only protected from cancer and exhibit extended longevity, but, according to the authors, they also present enhanced energy expenditure that participates in counteracting the development of obesity. This is related to lower BAT levels of phosphorylated AKT and FOXO1, higher BAT and WAT expression of UCP1, as well as of its transcriptional regulator, PGC1-α (Ortega-Molina et al., 2012). In addition, specific deletion of *Pten* in the liver in LPTENKO mice induces a strong hepatic steatosis (Stiles et al., 2004; Peyrou et al., 2015), but improves the overall insulin sensitivity, and decreases the fat mass. “Browning” of WAT could be one of the mechanisms underlying the increased insulin sensitivity of LPTENKO mice, in keeping with the observation of increased WAT glucose uptake (Peyrou et al., 2015). In humans, *PTEN* haploinsufficiency was shown to have divergent effects, as they increase the risk of obesity, while decreasing that of type 2 diabetes by markedly improving insulin sensitivity (Pal et al., 2012). In a very recent study, the grizzly bear was used as a hibernation model, in which obesity is a natural adaptation to survive months of fasting (Nelson et al., 2014). It was remarkably observed that preparation for hibernation was characterized by striking increases in body weight and in fat mass. Animals were shown to exhibit enhanced insulin sensitivity, while they become obese and to develop insulin resistance a few weeks later, during hibernation, to finally recover their insulin sensitivity upon awakening. The modification of insulin sensitivity occurs *via* the effect of the PTEN/AKT-mediated regulation of adipose tissue lipolysis. These results support the notion that adipose tissue is very insulin sensitive in the fed state, while being able to drive insulin resistance in the fasting state, independently from insulin levels (Nelson et al., 2014). In humans, the only physiological recovery of insulin sensitivity after a period of insulin resistance, partially due to an increase in food intake and lipogenesis, is observed in women after pregnancy (Barbour et al., 2007).

**Figure 1:**
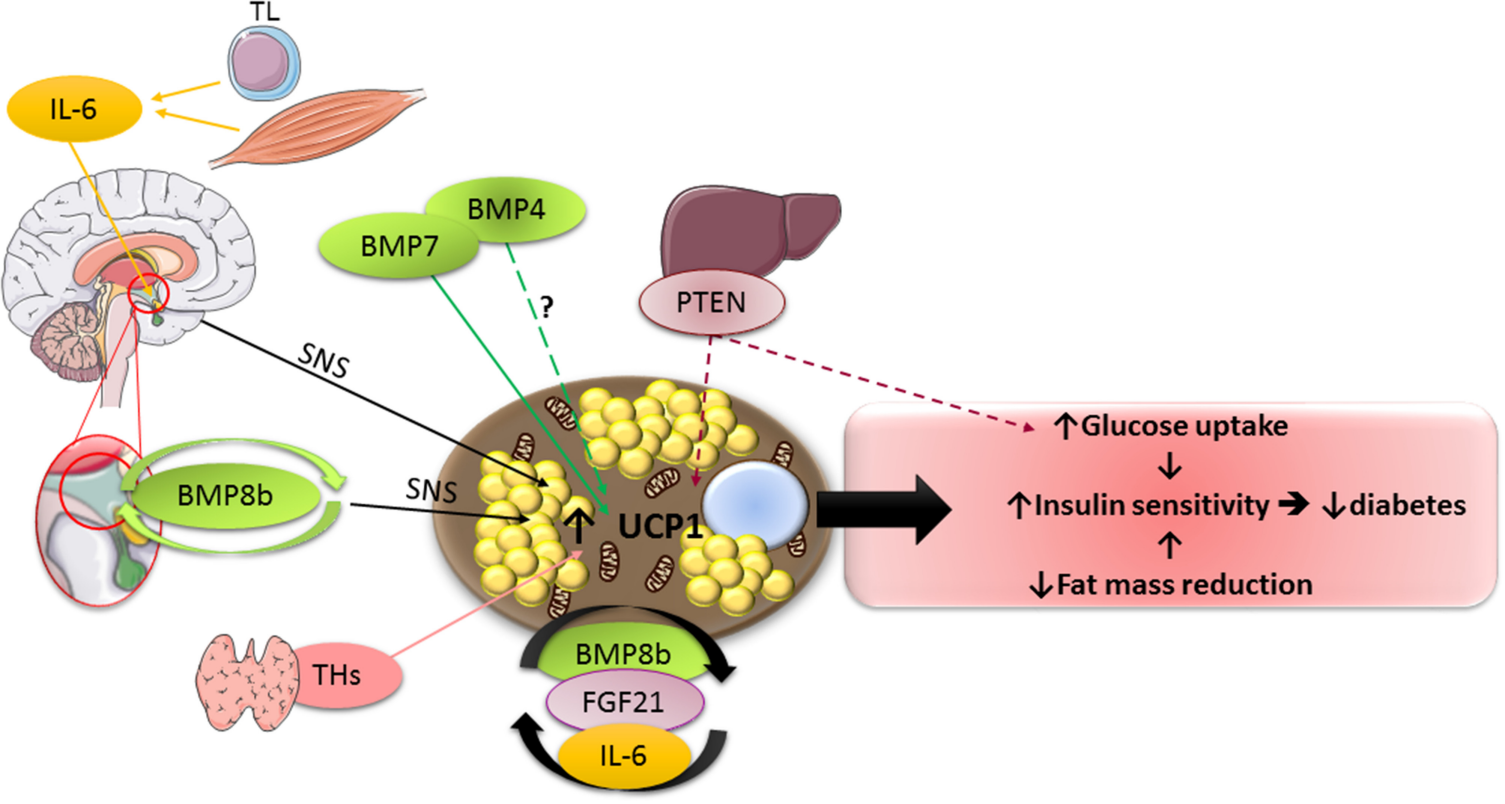
**Schematic representation of the main factors described in the review, which modulate UCP1 activity**. TL, T lymphocytes; SNS, sympathetic nervous system. For other abbreviations, see text. Full lines indicate stimulatory effects, whereas dotted lines represent inhibitory ones. ? indicates the existence of conflicting data in the literature with regard to the impact of the factor on UCP1 activity.

The present knowledge on the impact of the main batokines, as well as of the principal other UCP1 modulators on peripheral metabolism is schematized by Figure 1.

## Impact of perinatal nutritional changes

Epidemiological evidence in humans strongly suggests that the intrauterine and early postnatal environments have a significant long-term influence on body weight and energy homeostasis in offspring (for rev., see Breton, 2013). Thus, both maternal underfeeding or overfeeding were reported to exert a predisposing effect for the development of later obesity (Breton, 2013). Rodents are commonly used to investigate the mechanisms underlying long-term programming of energy balance in the offspring. High fat feeding of pregnant or lactating mothers was shown to induce glucose intolerance and the development of obesity in the progeny during adult life (Bayol et al., 2008; Samuelsson et al., 2008). The signals that mediate the effects of maternal metabolic disorders in overfed offspring have not been fully identified. They include hormones, such as insulin, leptin and glucocorticoids, proinflammatory cytokines, as well as complex epigenetic modifications (Tamashiro and Moran, 2010; Lukaszewski et al., 2013).

In this context, the sheep appears to be a very good model, because as for humans, the major source of BAT in the fetus is around central organs and is replaced by WAT after birth, whereas in rodents, the primary BAT depot is interscapular and it remains throughout life (Symonds et al., 2012). In sheep, the mother's diet during pregnancy determines the size of the placenta and can affect both the BAT and WAT mass, depending on the timing and the nature of the diet perturbation. In other words, the respective growth of the BAT and WAT depots depends on the maternal diet during gestation and it may be responsible for the development of obesity, insulin resistance and type 2 diabetes in the offspring, later in life (Symonds et al., 2012). Gestational diabetes in humans is a situation of increased nutrient supply that, together with the high maternal body mass index, can be accompanied by enhanced birth-weight and adverse long-term metabolic consequences, as described by the World Health Organization (2003), as well as in several publications (Larsson et al., 1986; Dabelea et al., 2000; Singh et al., 2006). However, in a recent study, the role of diabetes during gestation on such adverse long-term metabolic consequences has been seriously questioned, as they seem to relate more on known confounders, such as the BMI of either one of the parents (Donovan and Cundy, 2014).

Among the regulators that may link the maternal diet during gestation with the metabolic outcome of the offspring, leptin is one of the main candidates. It is a well-known hormone increasing BAT activity and inducing “browning” of WAT *via* activation of the sympathetic nervous system and resulting increased β3 adrenoceptor signaling (Haynes et al., 1997; Commins et al., 2000). Rodent models of leptin deficiency exhibit marked decreases in BAT thermogenic capacity, as well as activity (Ueno et al., 1998). Moreover, it was shown that leptin injection in the early stage of life in lambs decreases UCP1 expression in BAT, but improves thermoregulation, suggesting a particular role of leptin at such a stage in life in large mammals (Mostyn et al., 2002). Another study revealed that the ability of leptin to increase the metabolic rate early in life is independent from its anorectic action (Mistry et al., 1999).

No data are available as yet with regard to the impact of early leptin administration on the subsequent “browning” of WAT and the related regulation of glucose metabolism, as well as the response to hypercaloric diets later in life in the offspring.

## Attempts at stimulating BAT function in humans

With regard to the relationship between BAT and body weight in humans, an inverse correlation between BMI and the amount of BAT was described, already 5 years ago (Cypess et al., 2009; van Marken Lichtenbelt et al., 2009). In addition, more recent studies indicated that, compared to individuals without BAT, the BAT-positive subjects were younger, had lower body mass index, fasting insulin, insulin resistance, but a greater level of high-density lipoprotein cholesterol (Zhang et al., 2013). During acute cold exposure, BAT was shown as a significant independent determinant of plasma glucose and HbA1c levels (Matsushita et al., 2014). A parallel increase in BAT activity and cold-induced energy expenditure was also observed in response to acute cold exposure in subjects with low BAT activity, demonstrating the possible occurrence of BAT recruitment in humans (Yoneshiro et al., 2013). Very recently, chronic cold acclimation in human subjects was reported to increase the volume of metabolically active BAT, increasing its oxidative capacity, therefore its contribution to cold-induced thermogenesis (Blondin et al., 2014). In another study, the cold-induced increase in thermogenesis was accompanied by a decrease in body weight, mainly affecting the fat mass compartment (Yoneshiro et al., 2013). Cold-acclimation was also shown to increase diet-induced thermogenesis and postprandial insulin sensitivity, without impacting cold-induced thermogenesis (Lee et al., 2014b). These results are in keeping with data showing a physiological role of BAT in whole-body energy expenditure, glucose homeostasis, and insulin sensitivity in humans during prolonged cold exposure (Chondronikola et al., 2014).

Much more work is needed to identify other ways than cold exposure to increase BAT activity in obese subjects. To this end, one of the very useful tools is the use of rodent strains with different sensitivities to diet-induced obesity and insulin resistance. Indeed, resistance to the development of obesity has at least partly been attributed to elevated recruitment of brown adipocytes in skeletal muscle or WAT (Guerra et al., 1998; Almind et al., 2007; Veyrat-Durebex et al., 2009; Harms and Seale, 2013). These studies are highly therapeutically relevant, as BAT activation in overweight or obese subjects will activate thermogenesis and dissipate heat, while at the same time improving glucose metabolism and insulin resistance.

It should be added at that point that, although β3 adrenoceptors are expressed in humans (for rev., see Mund and Frishman, 2013) and β3 agonists are potent UCP1 activators in rodents, the molecules which are active in rodents cannot be used in humans due to inter-species differences. This should be solved in the future by the identification of selective human β3 agonists (Mund and Frishman, 2013; Bordicchia et al., 2014).

## Conclusion

To conclude, UCP1 is an excellent target to struggle diabetes and decrease body fat mass, improving whole metabolism. Indeed, it negatively regulates the energy balance by increasing energy expenditure. It also secretes several batokines, allowing for inter-organ communication. Finally, it is easily inducible, such as during mild cold exposure with resulting beneficial effects. Finding a molecule with as much efficiency as the β3 agonist in rodents, would be of considerable therapeutic relevance for the treatment of obesity, insulin resistance and type 2 diabetes in humans.

### Conflict of interest statement

The authors declare that the research was conducted in the absence of any commercial or financial relationships that could be construed as a potential conflict of interest.
